# Supplementary material for: Human Milk Feeding Is Associated with Decreased Incidence of Moderate-Severe Bronchopulmonary Dysplasia in Extremely Preterm Infants
Source: Children (Basel). 2023 Jul 23;10(7):1267. doi: 10.3390/children10071267 (PMC10378323; doi:10.3390/children10071267)
Supplement: Supplementary file 1 [file children-10-01267-s001.zip › children-2434771-supplementary.pdf]

Supplementary material

List of researchers and hospitals from the SEN1500 Neonatal Network

Gemma Ginovart (Hospital Universitari Germans Trias i Pujol). Josep Figueras Aloy; Francesc Botet Mussons (Hospital Clínic de Barcelona). Alberto Pérez Legorburu; Ana Gutiérrez Amorós (Hospital Universitario Basurto). Ramon Aguilera Olmos (Hospital General Universitario de Castellón). José Uberos Fernández (Hospital Universitario Clínico San Cecilio). Sandra Terroba; Santiago Lapeña (Hospital Universitario de León). Tomás Sánchez-Tamayo; Elías Tapia Moreno (Hospital Regional Universitario de Málaga). Dolores Elorza (Hospital Universitario La Paz). Araceli Corredera Sánchez; Enrique Criado Vega (Hospital Clínico San Carlos). Belén Fernández Colomer; José Enrique García López (Hospital Universitario Central de Asturias). María Ángeles Martínez Fernández (Complexo Hospitalario Universitario de Pontevedra). María Isabel de las Cuevas Terán; Ana Belén Pérez Santos (Hospital Universitario Marqués de Valdecilla). Miguel Ángel Cortajarena Altuna; Oihana Muga Zuriarrain (Hospital Universitario Donostia). Carmen Macias Díaz (Hospital Universitario Virgen del Rocío). Pedro Fuster Jorge; Mª Nieves González Bravo (Hospital Universitario de Canarias). Segundo Rite Gracia (Hospital Universitario Miguel Servet). Mª Purificación Ventura Faci; Mª Pilar Samper Villagrasa (Hospital Clínico Universitario Lozano Blesa). Mª Isabel Izquierdo Macián (Hospital Universitario y Politécnico de La Fe). Ana Belén Escobar Izquierdo (Hospital Virgen de la Salud - Complejo Hospitalario Universitario de Toledo). Alfonso Urbon (Hospital General de Segovia - Complejo Asistencial de Segovia). Mª Luz Couce Pico; María José Fernández Seara (Hospital Clínico Universitario de Santiago). Elena Pilar Gutiérrez González; Beatriz Vacas (Hospital Universitario de Salamanca - Complejo Asistencial Universitario de Salamanca). Manuel Baca Cots; Maribel Pérez Pérez (Hospital Quirónsalud Málaga). Dorotea Blanco Bravo; Susana Zeballos (Hospital General Universitario Gregorio Marañón). Mª Yolanda Ruiz del Prado; Inés Esteban Díez (Hospital San Pedro). Fermín García-Muñoz Rodrigo; Lourdes Urquía Martí (Hospital Universitario Materno-Infantil de Las Palmas GC). Rafael García Mozo; M. Teresa González Martínez (Hospital Universitario de Cabueñes). Mª Pilar Jaraba Caballero; Mª Dolores Ordoñez Díaz (Hospital Universitario Reina Sofía). Cristina de Frutos Martínez (Hospital Universitario de Burgos - Complejo Asistencial Universitario de Burgos). Sílvia Martínez-Nadal (SCIAS-Hospital de Barcelona). Martín Iriondo (Hospital Sant Joan de Déu). Amaya Rodríguez Serna; Lourdes Román Echevarría (Hospital Universitario de Cruces). María González Santacruz; Honorio Sánchez Zaplana (Hospital General Universitario de Alicante) María Fernanda

Moreno Galdo; José Antonio Hurtado Suazo (Hospital Universitario Virgen de las Nieves). Joan Badia Barnusell; Mònica Domingo Puiggròs (Corporació Sanitària Parc Taulí). Mª Mar Montejo Vicente; Raquel Izquierdo Caballero (Hospital Universitario Río Hortega). Aintzane Euba (Hospital de Txagorritxu - Hospital Universitario de Araba). Mar Albújar (Hospital Universitari Joan XXIII). Irene Cuadrado Pérez; Gema Villar Villar (Hospital Universitario de Getafe). Paula Serrano (Hospital Universitari Dexeus - Grupo Quirónsalud). Andrés Martínez Gutiérrez (Complejo Hospitalario Universitario de Albacete). Teresa Prada (Hospital El Bierzo). Elisenda Moliner Calderón (Hospital de la Santa Creu i Sant Pau). José Mª Barcia Ruiz (Hospital Infanta Margarita). María Jesús López Cuesta (Hospital San Pedro de Alcántara - Complejo Hospitalario Universitario de Cáceres). Mª José Santos Muñoz; Ersilia González Carrasco (Hospital Universitario Severo Ochoa). María Suárez Albo; Eva González Colmenero (Hospital Álvaro Cunqueiro - Complexo Hospitalario Universitario de Vigo). Víctor Manuel Marugán Isabel; Natalio Hernández González (Hospital Virgen de la Concha – Complejo Asistencial de Zamora). María Victoria Ramos Ramos; María Teresa de Benito Guerra (Hospital Universitario de Jerez de la Frontera). Gerardo Romera Modamio (Hospital Universitario HM Montepríncipe). Carolina Vizcaíno (Hospital General Universitario de Elche). Laura Acosta Gordillo; Josefina Márquez Fernández (Hospital Universitario Virgen de Valme). Alicia Mirada Vives (Hospital Universitario Mútua Terrassa). Eduard Soler Mir (Hospital Universitari Arnau de Vilanova). Mercedes Granero Asensio (Hospital Universitario Virgen Macarena). Javier Estañ Capell (Hospital Clínico Universitario de Valencia). Miguel Ángel García Cabezas; Mª Dolores Martínez Jiménez (Hospital General Universitario de Ciudad Real). Luisa López Gómez; Ana Campos Segovia (Hospital Universitario La Zarzuela – Sanitas). Alberto Trujillo Fagundo; Lluis Mayol Canals (Hospital Universitari de Girona Doctor Josep Trueta). Israel Anquela Sanz (Hospital de Granollers). Antonio Segado Arenas; Almudena Alonso Ojembarrena (Hospital Universitario Puerta del Mar). Carmen Rosa Pallás Alonso; María Teresa Moral Pumarega (Hospital Universitario 12 de Octubre). Sabina Romero; Mónica Rivero Falero (Hospital Universitario Nuestra Señora de Candelaria). Isabel Llana Martín (Hospital Universitario HM Torrelodones). Carmen González Armengod; Carmen Muñoz Labian (Hospital Universitario Puerta de Hierro). José María Lloreda García (Hospital General Universitario Santa Lucía). Laura Domingo Comeche (Hospital Universitario de Fuenlabrada). Laura Castells Vilella (Hospital Universitari General de Catalunya - Grupo Quirónsalud). María Pangua (Hospital Universitario de Guadalajara)
Concepción Goñi Orayen (Complejo Hospitalario de Navarra). Manuela López Azorín;. Adelina Pellicer (Hospital Universitario Quirónsalud Madrid). Mª Dolores Muro Sebastián;

Marta Balart Carbonell (Clínica Corachán). María González; José Enrique Sánchez Martínez (Vithas Hospital Parque San Antonio). Alejandro Ávila Álvarez; José Luis Fernández Trisac (Complexo Hospitalario Universitario de A Coruña). Paula Martín-Mora Bermúdez; Santos Olmedo San Laureano (Hospital Punta de Europa). Fátima Graña; Marta Padín Fontán (Complexo Hospitalario Universitario de Ourense). Emilia María Martínez Tallo (Hospital Materno Infantil - Complejo Hospitalario Universitario de Badajoz). David Lozano Díaz; Ana Muñoz Serrano (Hospital General La Mancha Centro). Isabel Llana; Mara Fernández Diaz (Hospital Universitario HM Puerta del Sur)

Lorena Patricia Peña González; Weimar García (Hospital Universitario Infanta Elena)

Elena García Victori; María Dolores Gómez Bustos (Hospital Quirónsalud Sagrado Corazón).
